# The relationship between structural campus climate and non-suicidal self-injury among middle school students: the chain-mediated roles of physical exercise and school bullying

**DOI:** 10.3389/fpsyg.2026.1760694

**Published:** 2026-03-09

**Authors:** Yang Zhou

**Affiliations:** Department of Physical Education, Xi'an Shiyou University, Xi'an, China

**Keywords:** chain mediation, non-suicidalself-injury, physical exercise, school bullying, structural campus climate

## Abstract

**Background:**

A structural campus climate (SCC) represents the organizational and orderly dimension of school climate. A high level of SCC means that a school possesses fair rules, reasonable value systems, and safety guarantees, all of which are key factors influencing adolescents' problem behaviors.

**Method:**

This study aimed to examine the relationship between SCC and non-suicidal self-injury (NSSI) among middle school students, and to analyze the chain-mediated roles of physical exercise (PE) and school bullying (SB). A total of 637 middle school students were surveyed using the Delaware School Climate Scale, Physical Activity Level Scale, School Bullying Scale, and Non-suicidal Self-injury Assessment Questionnaire.

**Results:**

SCC was and positively associated with PE, and SB was positively associated with NSSI. SCC and PE were each negatively related to SB; SCC and PE were also negatively related to NSSI. After controlling for factors such as gender, age, grade and only-child status, the mediation analysis indicated that the total effect of SCC on NSSI among middle school students was significant (β = −0.454, *P* < 0.001). The mediating effect values of PE and SB were −0.104 and −0.051, respectively. The chain mediating effect of PE and SB was significant, with a value of −0.048.

**Conclusions:**

PE and SB not only play independent mediating roles in the relationship between SCC and NSSI, but also function as sequential mediators in a chain-mediated pathway. The research findings underscore the importance of continuously optimizing the SCC in the intervention process for NSSI behavior among middle school students. On this basis, enhancing PE and reducing SB may provide effective avenues for alleviating NSSI behavior in this population.

## 1 Introduction

Non-suicidal self-injury (NSSI) refers to deliberate, direct, and socially unacceptable behaviors that cause harm to one's own body tissues without explicit suicidal intent, including actions such as cutting, scratching, and hitting (Wilkinson, 2013). As a dysfunctional emotion regulation strategy, NSSI has been widely recognized as a serious global public health issue (Mumme et al., 2017). Survey results indicate that the lifetime prevalence of NSSI among adolescents worldwide is as high as 22.1% (Lim et al., 2019); in China, the lifetime prevalence of NSSI among adolescents ranges from 15% to 41.5%, and it is showing an increasing trend year by year (Zhu et al., 2021). Research findings suggest that NSSI is significantly correlated with psychological disorders such as depression, anxiety, and substance abuse (Ghinea et al., 2019), and it also serves as a prospective predictor of suicidal behavior (Griep and MacKinnon, 2022). The Integrated Motivation-Volition Model posits (O'Connor et al., 2012) that adolescent self-injury behaviors occur through three stages: the pre-motivational stage, motivational stage, and volitional stage. Key environmental factors such as school and family atmosphere serve as core influencing elements during the pre-motivational stage of NSSI in adolescents. They not only constitute important contextual conditions that trigger such behavior but also represent critical entry points for interventions aimed at regulating self-injury risk. Schools, as the primary activity context for middle school students, expose this group to both intense academic competition and a heightened need for peer recognition and acceptance. Coupled with their still-developing cognitive capacities, adolescents are more prone to resort to NSSI as a maladaptive coping strategy when confronted with setbacks and difficulties (Espelage et al., 2003). Without timely and scientifically grounded interventions, such responses may precipitate more severe health-risk behaviors. In light of this, investigating the factors influencing NSSI among middle school students carries substantial practical significance for the prevention and intervention of mental health problems in this population.

### 1.1 SCC and NSSI among middle school students

As the primary setting for middle school students' daily activities, schools occupy a significant portion of their time and are the second most important ecosystem after the family (Hardcastle et al., 1981). Campus climate refers to the relatively enduring and stable environmental characteristics experienced by school members, which not only influence their psychological and behavioral outcomes (Hoy and Hannum, 1997) but also affect adolescents' externalizing problem behaviors (Tang et al., 2025; Li et al., 2015). Currently, academia categorizes campus climate into supportive campus climate and structural campus climate (Bear et al., 2011). The former includes teacher-student relationships and peer relationships, while the latter encompasses aspects such as school management, rule fairness, and school safety. Existing studies have shown that low-level SCC factors, such as the absence of campus disciplinary norms (Sharkey and Fenning, 2012) and imbalances in fairness (Burgess et al., 2023), can increase problematic behaviors among adolescents. Ecological Systems Theory suggests that schools, families, and individual characteristics are all micro-systems that influence adolescent development, and these ecosystems typically impact individual growth (Bronfenbrenner, 1979). This implies that when students perceive lower fairness and safety in the school environment, they are more likely to develop negative emotions (Lau et al., 2018), which may, in turn, increase their risk of engaging in NSSI as a maladaptive coping strategy. It is worth noting that existing research largely focuses on the role of SCC in Western contexts, while related studies in the Chinese context tend to concentrate more on the association between socially supportive school climate—such as peer relationships (Liu et al., 2022) and teacher-student relationships (Liu et al., 2025)—and NSSI among adolescents; few scholars have explored NSSI in middle school students from the perspective of SCC. Therefore, based on the aforementioned theoretical and empirical inferences, a lower level of SCC may be an important factor triggering individual NSSI. Accordingly, Hypothesis 1 is proposed: SCC negatively predicts NSSI among Chinese middle school students.

### 1.2 The mediating role of PE

Physical exercise (PE), as a culturally derived human activity, positively influences mood regulation and promotes physical and mental health (Brunet et al., 2013). Individuals who regularly engage in physical activities can strengthen their delayed gratification ability and persistence through goal-setting and achievement processes (Bai, 2003). In moderate-to-high-intensity physical activities, participants must overcome physical fatigue and psychological discomfort to achieve training goals, which serves as an effective way to cultivate patience, perseverance, and self-control (Mandolesi et al., 2018), while significantly reducing negative emotions such as depression, fear, and tension, thereby lowering the risk of risk behaviors driven by emotional dysregulation. A large body of research indicates that adolescents' self-control abilities (Huang et al., 2023) and emotion regulation (Zhou et al., 2025) are significantly negatively correlated with NSSI among adolescents. The Ecological Model of Physical Activity posits that individual exercise behavior is influenced by the interaction of individual and environmental factors (Spence and Lee, 2003), with the school environment being a crucial variable affecting participation in physical exercise (Ávila-García et al., 2025). Research indicates that high levels of SCC—characterized by clear goals, fair rules, and reasonable values (Stockard and Mayberry, 1992)—typically prioritize students' physical and mental health development, leading to greater investment in physical exercise resources and thus providing more opportunities for students to engage in such activities. Conversely, the absence of SCC may objectively limit students' enthusiasm for physical activities and subjectively weaken their willingness to participate (Dong and Mao, 2021), thereby reducing overall exercise participation rates. Based on relevant models and empirical studies, it is hypothesized that SCC may indirectly predict NSSI among middle school students through PE. Accordingly, Hypothesis 2 is proposed: PE has an independent mediating effect between SCC and NSSI in Chinese middle school students.

### 1.3 The mediating role of SB

School bullying (SB) refers to repeated, intentional aggressive behaviors in the school environment or school-related settings, where bullies inflict physical and emotional harm on victims, leading to maladaptive outcomes. Since this study utilizes the School Bullying Scale of the Olweus Bully/Victim Questionnaire for Children, which encompasses both the measurement dimensions of being bullied and engaging in bullying behavior, this study therefore focuses on an overall assessment of school bullying (Olweus, 1994). Existing research has identified school bullying as a significant potential factor contributing to NSSI among adolescents (Jiang and Chai, 2025). According to General Strain Theory, school bullying, as a major source of stress for students, can lead victims to develop negative self-cognition, such as self-denial and perceived personal deficiencies (Cole et al., 2016), which in turn elevate the risk of negative emotions like depression (Stubbs-Richardson and May, 2021). The accumulation of such negative emotions further exacerbates the risk of self-injury (Gollust et al., 2008). Additionally, a survey of 2,599 middle school students in China found significant negative correlations between students' perceptions of teacher-student relationships, clarity of expectations, and rule fairness, and their experiences of bullying (Xie and Mei, 2018). According to the Stage-Environment Fit Theory, healthy adolescent development requires trusting, supportive, and caring interpersonal relationships, as well as opportunities for self-expression, autonomous choice, and decision-making. When schools fail to provide these relational and autonomy-supportive conditions, a mismatch between the environment and individual developmental needs can lead to externalizing problems (Eccles and Roeser, 2012). When schools emphasize understanding and mastery in teaching, and when hardware facilities are better, the incidence of student aggression and victimization decreases (McEvoy and Welker, 2000). Conversely, the lack of clarity in school rules and weak enforcement often lead to an increase in both the incidence of bullying among students and the risk of victimization (Gottfredson et al., 2005). Based on the aforementioned relevant theories and empirical studies, it is hypothesized that SCC may indirectly predict NSSI among middle school students through SB. Accordingly, Hypothesis 3 is proposed: SB has an independent mediating effect between SCC and NSSI among Chinese middle school students.

### 1.4 The chain-mediated role of PE and SB

High levels of SCC may reduce the risk of NSSI among middle school students by increasing their participation in physical exercise, thereby decreasing the incidence of SB. Relevant studies have shown that high levels of SCC typically foster higher self-efficacy, lower school-related stress, and greater resilience (Wong et al., 2021), which optimize cognitive processes and influence behavioral selection, persistence, and the acquisition and performance of new behaviors (Yang, 2013). Research indicates that individuals with high self-efficacy are more likely to engage in persistent, moderately intense, and regular PE (Dong et al., 2013). Moreover, individuals who regularly participate in PE often establish higher-quality social relationships during sports, which may reduce the occurrence of SB. Additional studies have confirmed a significant correlation between SB and NSSI (Anan et al., 2025). Based on the aforementioned relevant theories and empirical research, it can be inferred that a positive SCC may encourage individuals to actively participate in PE, thereby reducing SB and ultimately lowering the risk of NSSI. Accordingly, Hypothesis 4 is proposed: PE and SB play a chain-mediated role in the relationship between SCC and NSSI among Chinese middle school students.

**Figure 1:**
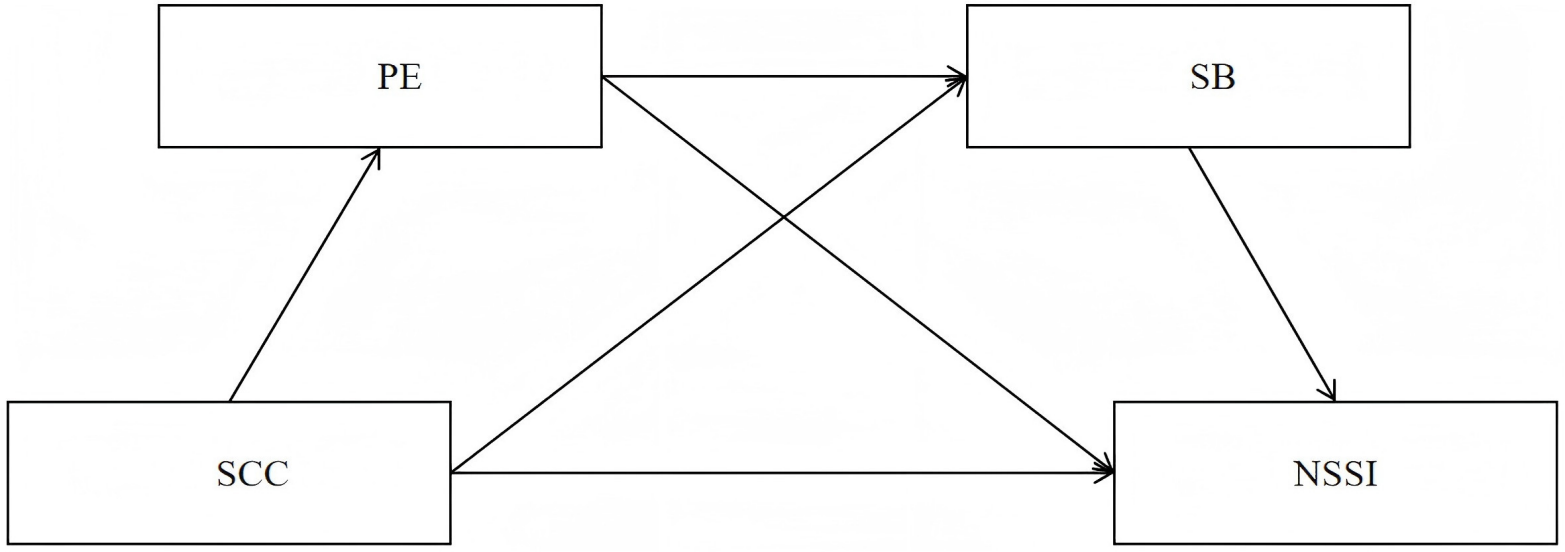
Hypothetical model diagram.

In summary, based on relevant theories and prior studies, this study aims to explore the relationship between SCC and NSSI, as well as the chain-mediated roles of PE and SB (see the hypothetical model in Figure 1). The findings aim to provide a novel perspective for preventing and reducing NSSI among middle school students.

H1: SCC negatively predicts NSSI among Chinese middle school students.
H2: PE has an independent mediating effect between SCC and NSSI in Chinese middle school students.
H3: SB has an independent mediating effect between SCC and NSSI among Chinese middle school students.
H4: PE and SB play a chain-mediated role in the relationship between SCC and NSSI among Chinese middle school students.

## 2 Methods

### 2.1 Participants

A convenience sampling method was used to test 700 students from three provinces in China. The selection was primarily based on covering the eastern, central, and western regions, while also taking into account differences in regional economic levels and the allocation of educational resources, which can effectively avoid the limitations of a single-region sample. The selected schools included both public and private institutions, as well as key and ordinary middle schools. This allows for reflecting differences in the construction of SCC under various educational models, thereby enhancing the generalizability of the research findings. Participants completed paper-based questionnaires under researcher guidance, yielding a total of 637 valid responses (valid response rate = 91.1%). The sample comprised 325 boys (51%) and 312 girls (49%). With respect to age, 487 participants (76.5%) were aged 12 years or younger, 115 (18.1%) were 13 years old, 32 (5.0%) were 14 years old, and 3 (0.5%) were 15 years or older. Regarding grade level, 441 participants (69.2%) were in the first year of junior secondary school, 116 (18.2%) in the second year, and 80 (12.6%) in the third year. As for sibling status, 126 participants (19.8%) were only children, and 511 (80.2%) had siblings.

### 2.2 Measurements

#### 2.2.1 Delaware school climate scale

The Chinese version of the Delaware School Climate Scale was employed (Ma, 2015); it comprises two principal components: supportive school climate subscale and structural school climate subscale (Bear et al., 2014). This study selects structural school climate subscale that represent the campus environment, including four dimensions: school engagement, clarity of expectations, fairness of rules, and campus safety. This scale contains 17 items, such as “The school's code of conduct is fair.” Responses were rated on a 4-point Likert scale ranging from 1 (strongly disagree) to 4 (strongly agree). The validity of this scale within the Chinese cultural context has been fully confirmed (Yuan et al., 2023). In this study, Cronbach's α was 0.926.

#### 2.2.2 Physical activity level scale

The Physical Activity Level Scale, the Chinese version revised by Liang (1994), was adopted. This scale assesses physical exercise across three dimensions: exercise intensity (e.g., high-intensity continuous activities such as running or swimming that induce shortness of breath and profuse sweating), exercise duration (e.g., 60 min or more), and exercise frequency (e.g., approximately once per day). Each dimension is scored on a 5-point scale (1–5 points). The total exercise score ranges from a maximum of 100 points to a minimum of 0 points. A score of 19 points or below indicates low physical activity, 20–42 points indicates moderate physical activity, and 43 points or above indicates high physical activity. Physical activity level is calculated as: Exercise Score = Frequency × (Duration – 1) × Intensity. The rationale for this formula lies in the standardized adjustment of the “Duration – 1” during the scale revision; the 1-point duration corresponds to “less than 30 min” (low activity duration), and subtracting 1 prevents the scores for low-level activity samples from approaching zero, while also aligning with the scoring intervals of the scale dimensions. Moreover, this formula has been widely applied and validated in studies involving domestic adolescent populations (Dong et al., 2025; Shang et al., 2023). In this study, Cronbach's α was 0.889.

#### 2.2.3 School bullying scale

The School Bullying Scale of the Olweus Bully/Victim Questionnaire for Children, revised by Zhang and Wu (1999), was used. The focus of this scale is on the overall incidence of school bullying, categorized into relational bullying, verbal bullying, and physical bullying, based on observation and perception. It does not specifically target the dimensions of being a victim or perpetrator of bullying. The scale consists of six items, with the specific dimensions and sample items as follows: Physical bullying dimension (e.g., “Some students hit, kick, push, or bump into other students or threaten them”), verbal bullying dimension (e.g., “Some students tease or mock other students”), and relational bullying dimension (e.g., “Some students deliberately isolate or exclude other students”). The scoring method uses a 5-point Likert scale, where 1 represents “Did not occur this semester” and 5 represents “Several times a week.” Participants are required to evaluate the frequency of bullying behaviors based on their observations or perceptions. In this study, Cronbach's α was 0.925.

#### 2.2.4 Non-suicidal self-injury assessment questionnaire

The Adolescent Non-suicidal Self-injury Assessment Questionnaire developed by Wan et al. (2018) was employed. This instrument comprises two sub-questionnaires: a behavior questionnaire and a functional questionnaire. In the present study, only the behavior questionnaire was used. It contains 12 items, such as “Deliberately pinching oneself,” and uses a five-point Likert scale ranging from 1 (never) to 5 (always). The validity of this scale has been fully verified within the context of Chinese culture (Xu et al., 2025). In this study, Cronbach's α was 0.972.

### 2.3 Procedure and data analysis

Data were analyzed using SPSS 26.0 for descriptive statistics, correlation analysis, and regression analysis. The mediation model was tested using the PROCESS macro developed by Hayes. The significance of the mediation effects was examined using the bias-corrected non-parametric percentile bootstrap method, and Model 6 provided by Hayes was selected for the current analysis.

## 3 Results

### 3.1 Common method bias test

To assess the potential presence of common method bias, the widely used Harman's single-factor test was employed. All 38 items from the four scales were entered into an unrotated exploratory factor analysis. The results indicated that four factors with eigenvalues greater than 1 were extracted. The first factor explained 33.30% of the total variance, which did not reach the commonly accepted threshold of 40%. Therefore, it was concluded that no serious common method bias was present in the present study. The systematic errors in the data collection process were minimal, and the research results are highly reliable.

### 3.2 Normality test of sample data

Descriptive statistics were used to analyze the variables. As shown in the table below (Table 1), the absolute values of kurtosis are less than 10, and the absolute values of skewness are less than 3. Therefore, all variables can be considered normally distributed.

**Table 1:** Descriptive statistics of variables.

**Dimensions**	**Number of cases**	**Minimum value**	**Maximum value**	**Average**	**Standard deviation**	Skewness		Kurtosis	
	**Statistics**	**Statistics**	**Statistics**	**Statistics**	**Statistics**	**Statistics**	**Standard error**	**Statistics**	**Standard error**
PE	637	0.00	100.00	46.096	30.050	0.236	0.097	−0.837	0.193
SCC	637	1.29	4.00	3.218	0.655	−0.894	0.097	0.355	0.193
SB	637	1.00	5.00	2.838	1.205	0.273	0.097	−1.116	0.193
NSSI	637	1.00	5.00	2.494	1.007	0.565	0.097	−0.459	0.193

### 3.3 Descriptive statistics and correlation analysis

Table 2 presents the intercorrelations among the key study variables. SCC was positively correlated with PE (β = 0.451, *P* < 0.01), indicating that the more complete the SCC, the higher the level of PE participation among middle school students. SB was positively correlated with NSSI (β = 0.350, *P* < 0.01), suggesting that the more frequent the SB, the higher the risk of NSSI among middle school students. Furthermore, SCC (β = −0.246, *P* < 0.01) and PE (β = −0.322, *P* < 0.01) were each negatively correlated with SB, meaning that good SCC and higher participation in PE can both reduce the occurrence of SB; similarly, SCC (β = −0.293, *P* < 0.01) and PE (β = −0.305, *P* < 0.01) were both negatively correlated with NSSI, indicating that better SCC and higher levels of PE can both lower the risk of NSSI.

**Table 2:** Descriptive statistics and correlation analysis.

**Variable**	**SCC**	***P***	**SB**	**NSSI**
SCC	1			
PE	0.451^**^	1		
SB	−0.246^**^	−0.322^**^	1	
NSSI	−0.293^**^	−0.305^**^	0.350^**^	1

^**^P < 0.01.

### 3.4 Mediating effect analysis

As shown in Table 3, the chain mediation of Model 6 was tested using the PROCESS macro in SPSS, with the Bootstrap sampling method (repeated 5,000 times). The mediating roles of PE and SB in the relationship between SCC and NSSI was analyzed while controlling for gender, age, grade level, and only-child status. The regression analysis results presented in Table 3 indicated that SCC had a significant positive predictive effect on PE (β = 20.783, *P* < 0.001). It should be noted that, according to the scoring rule for physical activity level defined as “Frequency × (Duration – 1) × Intensity,” the regression coefficient of SCC on PE is relatively large (β = 20.783). This is mainly because for each one-unit increase in the SCC, the students' physical exercise score increases by an average of 20.783 units. This corresponds to a comprehensive positive change in actual exercise behavior, such as an increase of 1 session per week in frequency, an extension of 30 min per session in duration, or a one-level increase in intensity, which is consistent with the scoring logic of the scale and the data distribution. SCC had significant negative predictive effects on NSSI (β = −0.252, *P* < 0.001) and SB (β = −0.235, *P* < 0.01). Meanwhile, PE had significant negative predictive effects on SB (β = −0.011, *P* < 0.001) and NSSI (β = −0.005, *P* < 0.001); SB had a significant positive predictive effect on NSSI (β = 0.216, *P* < 0.001).

**Table 3:** Regression analysis of chain mediation effect.

**Variable**	PE			SB			NSSI		
	β	**se**	***t***	β	**se**	***t***	β	**se**	***t***
Gender	−1.664	2.108	−0.789	−0.076	0.089	−0.852	−0.022	0.072	−0.312
Grade level	0.885	2.277	0.388	−0.009	0.096	−0.094	−0.003	0.077	−0.039
Age	−0.982	2.774	−0.354	0.172	0.117	1.469	0.076	0.094	0.806
Only-child status	1.926	2.693	0.715	0.006	0.113	0.049	0.036	0.091	0.394
SCC	20.783	1.634	12.721^***^	−0.235	0.077	−3.041^**^	−0.252	0.063	−4.019^***^
PE				−0.011	0.002	−6.364^***^	−0.005	0.001	−3.573^***^
SB							0.216	0.032	6.738^***^
R	0.452	0.352	0.432						
R-sq	0.205	0.124	0.187						
F	32.445	14.857	20.624						

^**^P < 0.01, ^***^P < 0.001.

As shown in Table 4, the results of the mediation effect test indicated that the mediating roles of PE and SB were significant, with a total mediation effect value of −0.202 and a total indirect effect accounting for 44.57%. This suggests that SCC can not only directly predict NSSI but also exert an indirect effect through the mediating pathways of PE and SB. Specifically, the mediating effects were transmitted through three mediation pathways: (1) The indirect effect 1 (−0.104) via the path “SCC → PE → NSSI,” with a Bootstrap 95% CI (LLCI = −0.170, ULCI = −0.040) that did not include 0, accounting for 22.80% of the total effect, indicating that SCC can indirectly reduce NSSI by promoting participation in PE; (2) The indirect effect 2 (−0.051) via the path “SCC → SB → NSSI,” with a Bootstrap 95% CI (LLCI = −0.089, ULCI = −0.018) that did not include 0, accounting for 11.19% of the total effect, indicating that SCC can indirectly reduce NSSI by lowering the incidence of SB; (3) The indirect effect 3 (−0.048) via the path “SCC → PE → SB → NSSI,” with a Bootstrap 95% CI (LLCI = −0.074, ULCI = −0.027) that did not include 0, accounting for 10.58% of the total effect, indicating that the pathway through which SCC enhance PE, subsequently reduce SB, and ultimately decrease NSSI is valid.

**Table 4:** Chain-mediated effect test.

**Variable**	**Effect**	**BootSE**	**BootLLCI**	**BootULCI**	**Total effect proportion**
Indirect effect 1	−0.104	0.033	−0.170	−0.040	22.80%
Indirect effect 2	−0.051	0.018	−0.089	−0.018	11.19%
Indirect effect 3	−0.048	0.012	−0.074	−0.027	10.58%
Total indirect effect	−0.202	0.037	−0.277	−0.134	44.57%
Direct effect	−0.252	0.063	−0.375	−0.129	55.43%
Total effect	−0.454	0.059	−0.569	−0.339	100%

## 4 Discussion

### 4.1 The relationship between SCC and NSSI among middle school students

This study found that SCC significantly and negatively predicts NSSI among middle school students, validating Hypothesis 1 and being similar to previous research (Holfeld and Baitz, 2020). More importantly, this finding supports and extends Ecological Systems Theory. Specifically, when schools provide a standardized, fair, and disciplined environment, they effectively meet middle school students' basic psychological needs for belonging, autonomy, and competence (Zumbrunn et al., 2014). This micro-system support is associated with the development of adolescent mental health, specifically manifested in that the enhancement of middle school students' subjective well-being, life satisfaction, and self-determination ability (Kleinkorres et al., 2023) is related to the accumulation of positive psychological capital; in turn, the accumulation of positive psychological capital is linked to the formation of adolescents' interpersonal interaction patterns and adaptive behaviors (Li and Dong, 2022), making this group less reliant on self-injurious behavior to seek emotional regulation or a sense of control, thereby potentially reducing the risk of NSSI. Conversely, when middle school students perceive a lack of fairness and safety in the school environment, they may develop negative self-cognition and value skepticism, along with rejection and doubt toward school norms (Emler and Reicher, 1987). Considering the sensitivity of adolescent development, individuals are more likely to develop negative perceptions of being rejected by their micro-systems, and these perceptions are associated with the occurrence of negative emotions such as depression and anxiety. When individuals lack adaptive resources in an imbalanced micro-system, NSSI may become an alternative means of coping with distress, potentially leading to an increase in NSSI behaviors. Thus, a positive SCC serves as a protective factor against NSSI among Chinese middle school students.

### 4.2 The mediating role of PE

This study found that PE mediates the relationship between SCC and NSSI among middle school students, validating Hypothesis 2. On the one hand, SCC significantly and positively predicts PE among middle school students, which is similar to prior research (Li et al., 2020). The Ecological Model of Physical Activity suggests that students' exercise levels are influenced by micro-environmental factors such as school sports facilities and regulations (Fang, 2010). A positive SCC requires clear teaching objectives, fair rules, and well-developed sports facilities (Yuan et al., 2023). Fair sports evaluation and reward mechanisms can enhance students' self-efficacy and sense of value, motivating them to participate in PE (Xin et al., 2006). Clear teaching objectives decompose complex skills into actionable steps, systematically improving students' motor skills and intrinsic motivation, thereby sustaining their exercise participation. Well-developed facilities lowers the barriers to exercise participation, supporting the development of stable exercise habits. The three elements are not isolated from one another; rather, they form a synergistic empowerment system with psychological motivation at its core, skill enhancement as the pathway, and hardware support as the guarantee, creating a closely linked and mutually supportive structure. This system helps promote the transformation of middle school students from passive participation to the active maintenance of exercise habits. On the other hand, PE significantly and negatively predicts NSSI among middle school students, which is similar to prior research (Yu et al., 2023). From the perspective of the intersection of exercise physiology and developmental psychology, the increase in neurotransmitters such as dopamine and endorphins in the blood varies depending on the intensity and duration of physical exercise (Chaouloff, 1989). Among these, moderate-intensity exercise of appropriate duration provides the most effective stimulation (Alves et al., 2014), helping the stress hormone cortisol levels return to normal (Lou et al., 2023). This directly interrupts, from a physiological standpoint, the transmission chain in which accumulated negative emotions lead to the pursuit of extreme release. It is worth noting that middle school students are in adolescence, and their emotional regulation abilities are not yet fully developed. NSSI is often seen as a passive alternative for coping with negative emotions. PE, through the balance of neurotransmitters at the physiological level, fosters the enhancement of positive emotions and the alleviation of negative ones, thereby reducing middle school students' tendency to rely on NSSI to regulate their emotions.

### 4.3 The mediating role of SB

This study found that SB mediates the relationship between SCC and NSSI among middle school students, validating Hypothesis 3. On one hand, SCC significantly and negatively predicts SB among middle school students, which is similar to prior research (Zhang et al., 2021) and validating the Stage-Environment Fit Theory. In this study, a positive SCC provides clear, fair disciplinary norms, explicitly defines bullying behaviors and their consequences (Wen and Chen, 2020), and makes potential bullies aware of the severe repercussions of such actions, thereby deterring bullying. Additionally, integrating anti-bullying education into school curricula helps students establish healthy habits and rational value systems, reducing bullying incidence (He et al., 2025). Conversely, if the SCC fails to provide a positive experience, middle school students may withdraw from real-life interpersonal interactions and shift their focus to bullying. For example, Kidger's longitudinal study showed that middle school students lacking school belongingness or perceiving teacher unfairness were more likely to engage in NSSI two years later (Kidger et al., 2015). On the other hand, SB significantly and positively predicts NSSI among middle school students, which is similar to prior research (Jing et al., 2025). When exposed to physical attacks, verbal abuse, or other forms of bullying, middle school students often experience anxiety and worry (Wu and Zhang, 2024), leading to rumination—a repetitive focus on negative thoughts (Monti et al., 2017). This cognitive pattern can, to some extent, continuously amplify the intensity and duration of negative emotions, thereby reinforcing an individual's urgent motivation to escape aversive experiences. NSSI, as an easily implementable attention-shifting strategy, can guide adolescents to redirect their focus from negative thoughts to intense bodily sensations, thus quickly regulating negative emotions (Selby and Joiner Jr, 2009). This short-term alternative regulatory pathway further strengthens the association between self-injury and emotional relief, thereby promoting the occurrence of NSSI.

### 4.4 The chain-mediated role of PE and SB

This study found that PE and SB play a chain-mediated role in the relationship between SCC and NSSI among middle school students, validating Hypothesis 4. Regular participation in PE is associated with improvements in the physical fitness and health levels of middle school students and is often accompanied by enhanced physical coordination and strength. This physical capital not only serves as a foundation for physical protection but also translates into psychological self-efficacy and a sense of boundaries, enabling students to face potential bullying with confidence, to clearly refuse and seek help rather than passively endure, thereby reducing the likelihood of victimization. Furthermore, engaging in PE requires adherence to rules, emotional regulation, and cooperative interactions aimed at mutual success. Such contextualized experiences systematically cultivate self-control (Wang et al., 2025), effectively regulating impulsive and aggressive tendencies, and reducing the motivational drivers for bullying that arise from emotional loss of control or attempts to assert power. Empirical research mentioned early shows that a positive SCC, by providing clear exercise goals, safe environments, and adequate resources, lowers participation barriers and encourages sustained exercise habits. Exercise, in turn, reduces bullying through behavioral shaping and emotional regulation, thereby mitigating negative emotion accumulation and self-injury motives, ultimately preventing NSSI among middle school students.

## 5 Recommendations

Based on the findings, the following strategies are proposed to prevent and intervene in NSSI among Chinese middle school students:

(1) Establish clear rules to define behavioral boundaries: Schools should formulate Graded Disposal Guidelines for School Bullying, clarifying the definition criteria for bullying behaviors such as verbal humiliation, physical conflict, and social isolation, and establishing a mechanism of anonymized student reporting and 24/7 teacher response. Meanwhile, a joint screening mechanism for bullying - rumination - self-injury should be constructed. Combining the School Bullying Scale, Rumination Scale, and NSSI Behavior Questionnaire, several rounds of anonymous questionnaire screenings should be conducted each semester to identify high-risk groups. Individualized tracking and management files should be established, with psychological teachers regularly assessing students' emotional states. To help bullying victims break the cycle of negative thinking, group counseling on “thought stopping + emotion regulation” should be launched for them, including modules such as negative thought identification and problem-solving skill drills, so as to assist students in breaking the cycle of negative thinking. To accommodate schools with different accommodation models, boarding schools can implement this during evening self-study sessions or weekend stays, with the module supplemented by techniques for dealing with interpersonal conflicts in dormitories; non-boarding schools can incorporate it into after-school service periods, simultaneously providing parents with simplified methods for emotional guidance to extend the effectiveness of counseling.
(2) Foster a supportive campus climate through fairness and safety: At the equity level, transparent mechanisms for resource allocation and disciplinary enforcement should be established to ensure procedural justice in areas such as distribution of teaching resources and selection for honors and awards, thereby alleviating students' hostility and sense of alienation stemming from perceived unfairness. At the safety level, a comprehensive prevention and control system should be relied upon. This can be achieved by strengthening patrol supervision, promptly intervening in potential conflicts (e.g., physical clashes, verbal humiliation), and complementing these measures with systematic safety protection education, so as to effectively safeguard students' personal and psychological safety and enhance their self-protection capabilities.
(3) Implement specific physical education programs to enhance students' emotion regulation and self-protection abilities. Launch collective micro-exercise during the 10-min break between classes: PE teachers uniformly design diverse activity contents (e.g., jumping jacks + simple stretching + collective clapping exercises) and produce teaching videos to distribute to each class; head teachers organize the activity daily during breaks, requiring full participation, with sports committee members taking charge of leading the exercises to ensure the exercise duration per break is no less than 8 min. Meanwhile, PE teachers and psychological teachers co-teach courses divided into several thematic modules, such as jogging for stress reduction, ball game confrontations for emotional release, and mindfulness yoga for anxiety relief. Each class includes three parts: emotional self-assessment (5 min), specialized exercise (30 min), and sharing and communication (10 min), aiming to regulate students' emotions through physical activity.

## 6 Limitations

(1) All data were collected via student self-reports, which may be susceptible to common method bias. Future studies could integrate objective methods such as neurophysiological measurements and behavioral experiments to more deeply reveal the formation mechanism of NSSI among middle school students. Meanwhile, this study used self-reported scales to collect exercise-related data, which may be plagued by issues such as recall bias (e.g., underestimating or overestimating exercise duration and frequency) and subjective embellishment of exercise behaviors. These limitations make it difficult to accurately capture the actual implementation of exercise (e.g., true intensity, whether it was interrupted midway). This measurement constraint may to a certain extent affect the accuracy of the mediating effect of PE; future studies could further verify this using objective measurement tools such as fitness trackers and heart rate monitoring.
(2) This study mainly focuses on the SCC and failed to fully incorporate important interpersonal factors such as family environment and teacher-student relationships. Future research could build a more comprehensive explanatory framework by integrating multi-level variables based on the ecological model.
(3) This study employed a cross-sectional research design, which limits causal inference. Although the proposed pathway is theoretically plausible, cross-sectional data cannot rule out the possibility of other reverse causal relationships: for instance, students with a lower incidence of NSSI may possess more positive psychological states and interpersonal adaptability, and such students may be more inclined to actively engage in PE. This limitation precludes the current study from clarifying the dynamic order of action among the variables.
(4) This study employed a convenience sampling method. While this approach facilitates data collection and enhances research feasibility, the selection of samples depends on the cooperation of schools, which may introduce sampling bias. Furthermore, the participating schools are mainly concentrated in urban provinces, failing to fully represent the national middle school student population; therefore, the generalizability of the study findings warrants careful consideration. In the future, a stratified random sampling method could be used to expand sample coverage based on a three-dimensional framework of “region, school type, and educational stage,” thereby improving the applicability of the research.

## 7 Conclusions

PE and SB not only respectively play independent mediating roles between the SCC and NSSI among middle school students, but also together form a serial mediating pathway. Specifically, by creating a SCC characterized by fair rules and safety guarantees, it is possible to directly mitigate the risk of self-injury among middle school students; it can also reduce NSSI by enhancing students' participation in PE, thereby improving their emotional regulation and self-control abilities; additionally, it can indirectly interrupt the pathways that trigger self-injurious behaviors by lowering the occurrence of SB. More importantly, a positive SCC can further reduce the incidence risk of NSSI among middle school students through the serial transmission of improving PE participation → reducing SB incidence. Furthermore, the generalizability of the conclusions of this study should be limited to populations with characteristics similar to the study sample and should not be overextended to all adolescent groups. Future research could consider expanding the sample size and employing longitudinal tracking design and mixed-method approaches to further verify the causal relationships among the variables “SCC, PE, SB and NSSI,” thereby enhancing the external validity and explanatory power of the research findings.

## Data Availability

The original contributions presented in the study are included in the article/Supplementary material, further inquiries can be directed to the corresponding author.
